# Adipose-Derived Stem Cells as Carrier of Pro-Apoptotic Oncolytic Myxoma Virus: To Cross the Blood–Brain Barrier and Treat Murine Glioma

**DOI:** 10.3390/ijms252011225

**Published:** 2024-10-18

**Authors:** Joanna Jazowiecka-Rakus, Kinga Pogoda-Mieszczak, Masmudur M. Rahman, Grant McFadden, Aleksander Sochanik

**Affiliations:** 1 Center for Translational Research and Molecular Biology of Cancer, Maria Skłodowska-Curie National Research Institute of Oncology, Gliwice Branch, Wybrzeże AK 15, 44-102 Gliwice, Poland; kinga.pogoda-mieszczak@gliwice.nio.gov.pl; 2 School of Life Sciences, Arizona State University, Tempe, AZ 85281, USA; masmudur.rahman@asu.edu (M.M.R.); grantmcf@asu.edu (G.M.); 3 Center for Personalized Diagnostics, Biodesign Institute, Arizona State University, Tempe, AZ 85281, USA

**Keywords:** myxoma virus, oncolytic virotherapy, adipose tissue-derived stem cells (ADSCs), glioblastoma, blood–brain barrier

## Abstract

Treatment of glioblastoma is ineffective. Myx-M011L-KO/EGFP, a myxoma virus actively inducing apoptosis in BTICs linked to recurrence, offers innovative treatment. We loaded this construct into adipose-derived stem cells (ADSCs) to mitigate antiviral host responses and enable systemic delivery. The apoptotic and cytotoxic effects of the construct were studied using murine and human glioblastoma cell lines. Before implementing systemic delivery, we delivered the construct locally using ADSC to verify elimination of orthotopic murine glioma lesions. vMyx-M011L-KO/EGFP was cytotoxic to a murine cell line, preventing effective virus multiplication. In three human glioma cell lines, viral replication did occur, coupled with cell killing. The knock-out construct induced apoptotic cell death in these cultures. ADSCs infected ex vivo were shown to be sufficiently migratory to assure transfer of the therapeutic cargo to murine glioma lesions. Virus-loaded ADSCs applied to the artificial blood–brain barrier (BBB) yielded viral infection of glioma cells grown distally in the wells. Two rounds of local administration of this therapeutic platform starting 6 days post tumor implantation slowed down growth of orthotopic lesions and improved survival (total recovery < 20%). ADSCs infected ex vivo with vMyx-M011L-KO/EGFP show promise as a therapeutic tool in systemic elimination of glioma lesions.

## 1. Introduction

Glioma, the most common brain tumor, arises from glial cells. The most malignant form, glioblastoma multiforme (GBM), with an annual incidence of almost 300,000 cases worldwide and a dim prognosis, remains a therapeutic challenge [1,2]. The recurrence of GBM after surgical resection, leading to its high mortality, is caused by cancer stem cells termed brain tumor initiating cells (BTIC). The glioma tumor microenvironment tends to be immunosuppressive, promoting its proliferative potential and invasiveness. Together with immune evasion mechanisms, these features make glioma a difficult therapeutic challenge. Despite decades of research and clinical trials, progress in treatment remains very unsatisfactory [3]. New strategies are needed to eliminate malignant glioma cells in situ, particularly the stem-like cancer cells that resist most other therapies.

Oncolytic therapy is on the forefront of innovative approaches to glioma treatment. It explores specific types of viruses capable of selective infection and elimination of cancer cells [4]. Oncolytic viruses (OVs) directly kill tumor cells, activating a robust inflammatory response that promotes antitumor immunity. Also, genetic modification of OVs with various transgenes may add to their therapeutic potency; for example, by boosting the anti-tumor immune response of the cancer-burdened host. We here used a specific recombinant construct of myxoma virus (MYXV), an enveloped double-stranded DNA virus from the *Poxviridae* family. MYXV is a potent oncolytic agent, nonpathogenic for humans and rodents, capable of productively infecting many classes of human cancer cells and has previously shown potential effectiveness against glioma, medulloblastoma, melanoma, pancreatic cancer, osteosarcoma and myeloma [5,6,7,8,9,10,11].

The myxoma virus construct used here can be particularly useful in glioma studies since it can pro-actively trigger apoptosis of BTIC, brain tumor initiating cells, i.e., glioma stem cells deemed responsible for inevitable glioma recurrence. Thus, this MYXV construct is an important candidate for clinical translation [5]. BTICs are intrinsically resistant to apoptosis due to high expression of antiapoptotic proteins and sensitizing these cells to apoptosis would enhance the efficacy of the oncolytic strategy. Apoptosis is a highly efficient mechanism of viral evasion since it activates effector caspases that shut down downstream transcription and translational machinery, preventing it from being hijacked by replicating viruses. To circumvent this, many viruses encode anti-apoptotic proteins. M011L, one of these apoptosis blockers, inhibits the programmed cell death response to viral infection by sequestering Bak and Bax and inhibiting cytochrome c release. An MYXV construct lacking the *M011L* gene (vMyx-M011L-KO/EGFP) was shown to trigger apoptosis in infected BTICs. Following infection, activation of host proapoptotic Bcl-2 proteins initiates mitochondrial events, culminating in the activation of effector caspases, which prevents transcription and translation from takeover by replicating viruses [5]. However, intratumoral delivery of such a therapeutic to glioma lesions in situ is technically limited and sometimes simply impossible.

On the other hand, systemic delivery of oncolytic viruses is restricted by the presence of the blood–brain barrier (BBB). The BBB is a physical barrier formed by capillary endothelial cells. The main components of the junctions are transmembrane proteins, namely occludin and a family of endothelial proteins (claudins). In addition, the barrier functions owing to the membrane lipid bilayers of the capillary endothelium, efflux transporters, and supporting cells such as pericytes and astrocytes. The BBB is the most tightly regulated gateway in the human body, fed by 100 billion blood capillaries. It is routinely capable of excluding most therapeutics. This selective barrier forms an interface of highly specialized brain microvascular endothelial cells regulating the transport of ions, hydrophilic nutrients, and metabolites, as well as required macromolecules to help maintain proper brain function, while also excluding toxins from the central nervous system [12,13]. The BBB excludes the majority of small and large molecule drugs from entering the brain. The endothelial cells forming the BBB lack fenestrae, possess solute carriers that regulate ion and small molecule transport, and express efflux transporters and receptor-mediated processes for specific uptake of macromolecules. The complexity of the BBB creates major obstacles for brain drug delivery and is the primary cause of treatment failure. To facilitate screening drugs for BBB permeability, in vitro models of an artificial BBB have been developed, making use of brain endothelial cells cultured on extracellular matrix-coated porous inserts [14,15]. 

Besides the presence of the BBB, systemic delivery of oncolytic viruses is restricted also by innate antiviral host responses. Carriers protecting the viral cargo during blood transit, as well as capable of overcoming the BBB, would be necessary to attempt successful systemic OVs delivery to glioma lesions. Mesenchymal stem cells (MSCs) carrying viral particles evade detection by antibodies and T cells since stem cell antigen processing transporters are low in number. In vitro and in vivo studies have shown the susceptibility of stem cells to viral infection and replication, as well as their intact migratory tumor tropism. The use of stem cells as a stealth-type carrier for in vivo transfer of cancer therapeutics has shown much promise for a range of tumor models [16]. Considering that delivery to the brain should be minimally invasive, repeatable and devoid of neurosurgery pitfalls such as hemorrhages and infection, MSC-mediated transport using a “Trojan horse” strategy offers high therapeutic agent loading capacity and diminished immunogenicity, targeted delivery, controlled drug release and prolonged drug survival. 

The tight junctions (ca. 1 µm in size) between endothelial cells are structurally dynamic. Brain cancer cells secrete vascular endothelial growth factor (VEGF), resulting in the disappearance of astrocyte endfeet and the disruption of tight junctions and the appearance of paracellular fenestrations [17]. Brain tumor microenvironment-secreted cytokines leak into the bloodstream through the fenestrations of the capillary endothelial cell layer at the BBB and can attract MSCs to the brain lesion. MSCs are thought to migrate across the BBB to tumor foci in the brain in a manner similar to that used by cancer cells metastasizing from peripheral tumor foci. MSCs cross the BBB by passing through transiently formed fenestrations between vascular endothelial cells; due to the chemoattractant gradient of cytokines secreted in the tumor microenvironment and leaking into the bloodstream through the fenestrations, MSCs can reach their target. Anticancer drugs, on the other hand, penetrate the brain through paracellular fenestration and transcellular pinocytosis [17].

Of the various anticancer cargoes that have been loaded into MSCs, oncolytic viruses are among the most promising for the treatment of brain tumors [18]. We here examined in vitro the transit of ADSCs loaded ex vivo with a proapoptotic oncolytic myxoma construct (vMyx-M011L-KO/EGFP) through an artificial BBB. Before attempting systemic delivery of this therapeutic platform in vivo, we first demonstrated its potential for the elimination of experimental orthotopic glioma lesions in mice via local administration. 

## 2. Results

### 2.1. Oncolytic vMyx-M011L-KO/EGFP Construct Destroys Glioma Cell Cultures

**Figure 1:**
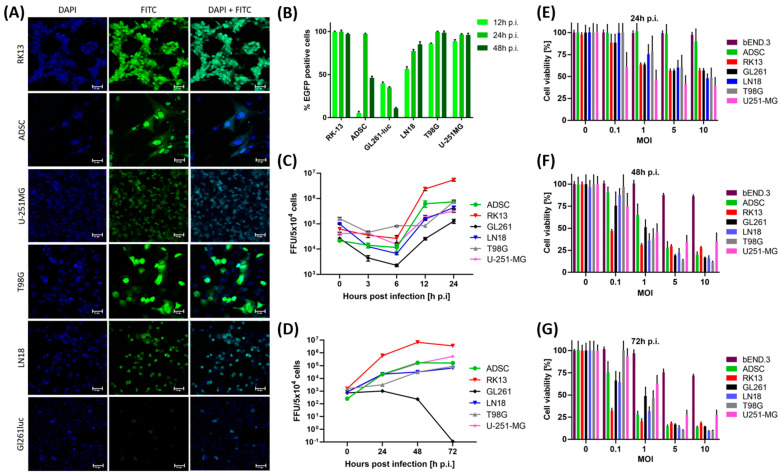
Permissiveness and cell viability. Cell lines: RK-13 and ADSCs as well as GL261, T98G, LN18, and U-251MG (gliomas) infected with vMyx-M011L-KO/EGFP; (**A**) Representative images of infected cells (MOI = 5) after 24 h p.i. stained and visualized by fluorescence microscopy (Zeiss LSM 710 confocal workstation; magn. 20×; scale bar = 50 µm); blue: DAPI dye (nuclei); green: EGFP fluorescence; (**B**) Flow cytometric quantitation (12–48 h p.i.) of infected EGFP-positive cells in tested cell lines (MOI = 5); Replication of the virus in tested cell lines by determining (**C**) the single- (MOI = 5) and (**D**) multi-step virus growth curve (MOI = 0.02). Cell viability assay (MTS) for bEnd.3, RK-13, ADSCs and glioblastoma GL261, T98G, LN18 and U-251MG lines and four different MOI values: (**E**) 24 h, (**F**) 48 h and (**G**) 72 h time points p.i. and eight independently infected wells were examined. Error bars represent SD of the measurements.

The in vitro infectivity of the vMyx-M011L-KO/EGFP construct (multiplicity of infection, MOI = 5) was assessed using cell cultures of the GL261luc, LN18, T98G and U-251MG glioma lines as well as the RK-13 and ADSC lines as controls. Microphotographs (Figure 1A) demonstrate EGFP fluorescence of the infected cell cultures. Microscopic data confirm rather abundant EGFP expression in the infected cultures of LN-18 and T98G human gliomas (as well as in RK-13 and ADSC), whereas the fluorescent protein signal in the infected murine glioma cell line (GL261) was incomparably lower compared to its human counterparts. The time-lapse data of early infection showed GL261 cells killed by the virus within the initial 5–12 h period post infection (h p.i.), leaving only single live and infected cells after 24 h (Video S1: MYXV infection in vitro kills glioma cells in culture).

Flow cytometry histogram data (Figure 1B) show the percentage of EGFP-positive infected cells. The data reveal varying EGFP cell levels depending on the examined cell line and time point after infection. For the RK-13 (MYXV propagation) line, the percentage of EGFP-positive population reached 96% after 12 h p.i. and remained at that level for up to 72 h. For the LN-18, T98G, and U-251MG human glioma lines, the percentage of EGFP-positive cells increased over time, reaching a maximum after 48 h (85%, 98%, and 95%, respectively). Infection of the murine cell line GL261 was low (39%) and decreased over time with the percentage of EGFP-positive cells dropping to 10% after 48 h, which indicates a lack of virus multiplication. A contrasting EGFP expression pattern was observed for ADSCs (viral carrier): with 5% percent of EGFP-positive cells at 12 h p.i. to 97% after 24 h only to decrease to 46% after 48 h. 

These results show that RK-13, ADSCs, and human gliomas are permissive toward the myxoma construct, which multiplies effectively in the cytoplasm to produce functional progeny virions, with the number of viral copies similar for individual cell lines. GL261, the murine glioma cell line, appears as semi-permissive and exhibited different EGFP expression characteristics upon infection with a myxoma knockout construct. The input virus penetrates cells, as visualized by fluorescence microscopy (Video S1), but is unable to multiply effectively, presumably due to the strong and rapid cytotoxic effect of the vMyx-M011L-KO/EGFP construct. 

We analyzed the replication of the vMyx-M011L-KO/EGFP virus in the individual cell lines by generating single-step and multi-step growth curves. Single-step growth curves were performed at high MOI to assess the infectious progeny produced during a single myxoma virus replication cycle (Figure 1C). The cell lines were infected with vMyx-M011L-KO/EGFP (MOI = 5) and samples were taken to assess the infectious virus particles at 0–24 h post-infection by titrating the RK-13 cells via serial dilution. The single-step growth curves obtained resembled the classical varicella virus replication curve, reaching a nadir after about 6 h of infection, followed by a continuous increase up to 24 h p.i., at which point the maximum viral yield was reached. The highest replication efficiencies were obtained for the RK-13 cell line whereas the lowest were obtained for GL261-luc. Multi-step growth curves were generated at a lower MOI (0.02) and for longer periods (up to 72 h p.i.) to quantify multiple rounds of virus replication and assess cell-to-cell spread (Figure 1D). Infected cell lines were harvested for infectious virus particles at 0–72 h after infection. The samples were titrated on RK-13 cells by serial dilution. Myxoma virus successfully underwent several rounds of replication and cell-to-cell progression staggered over time as seen by the increasing viral load for all cell lines tested except GL261-luc (Figure 1D). In this cell line, following infection with vMyx-M011L-KO/EGFP the virus did not multiply; infected cells became rounded and presumably were destroyed before the 24 h time-point p.i. (Video S1). The GL261-luc cell line was thus found to be susceptible but semi-permissive for MYXV. In contrast, the results showed that ADSCs allowed and supported both single and multiple rounds of myxoma virus replication.

We examined the effect of myxoma virus construct infection on cell viability of RK-13, ADSCs, bEnd.3 and glioblastoma cell lines by performing MTS cell assays using four different MOIs (0.1, 1, 5 and 10) and three time points (24, 48 and 72 h) p.i. (Figure 1E–G). For MOI 5 and 10, we found no major differences among the tested cell lines. At MOI = 5, the cytotoxic effect of the MYXV construct in RK-13, GL261-luc, LN-18 and T98G cell lines after 24 h caused viability to decrease to ~56–60%, and after 72 h to 18 %, 16%, 13% and 10%, respectively. The least susceptible cell line was U251-MG, which after 24 h was found to be 45% viable at MOI = 1 and 41% viable at MOI = 5; after 72 h, it was 62% viable at MOI = 1 and 27% at MOI = 5. 

We observed high survival of ADSCs after 24 h p.i.: 100% for MOI = 1, 98% for MOI = 5, and ~90% for MOI = 10 (Figure 1E). After 48 h p.i., the number of viable cells decreased to 65% for MOI = 1 and to 27% for MOI = 5 (Figure 1F). However, after 72 h p.i., the number of viable ADSCs was 14% (Figure 1G). Thus, the ADSC cell line, of interest as a viral carrier for in vivo therapy, did demonstrate a high percentage survival for the 24 h time interval p.i., which is more than satisfactory as a therapeutic window level.

The bEnd.3 cell line was found to be non-permissive to the MYXV and showed 100% viability after 24 h p.i. regardless of the MOI used. Viability of 87% was observed for MOI 5 and 10 after 48 h p.i., and 71% at 72 h p.i.

### 2.2. Myxoma Virus Construct Induces Apoptosis

Apoptosis was identified using Annexin V binding to phosphatidylserine, which migrates to the outer cell membrane. Subsequent cell membrane perforation allows for distinguishing early and late apoptosis, as well as necrosis, using 7-aminoactinomycin (7-AAD) dye penetration into the cell through damaged membranes. 

**Figure 2:**
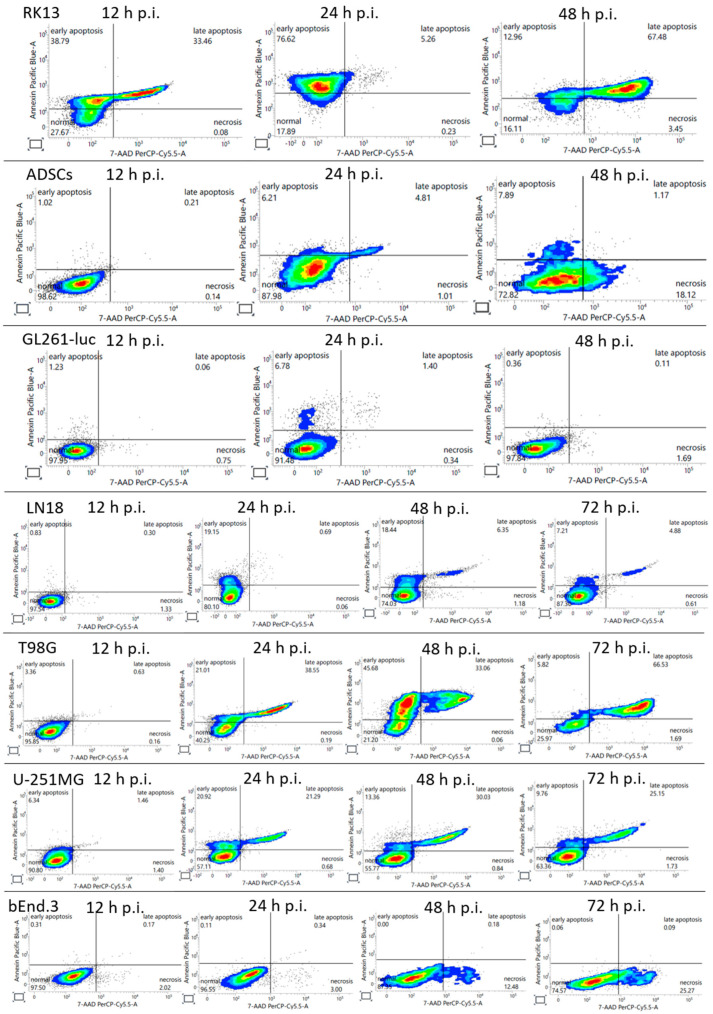
Analysis of Apoptosis by Flow Cytometry. Cells were seeded at a density of 1 × 10^5^ cells/well using 6-well plates and vMyx-M011L-KO/EGFP (MOI = 5) was added to the cultured cells. After 12, 24 h and 48 h (RK-13, ADSCs, and GL261-luc) and after 12, 24, 48 and 72 h p.i. (LN18, T98G, U-251 MG and bEnd.3) cells were collected and washed twice with PBS^−^ and staining buffer. Cells were then stained with Annexin V-Pacific Blue™ and 7-AAD and analyzed for apoptosis/necrosis using flow cytometry (BD FACSLyric™). Annexin V was detected using the Pacific Blue channel and 7-AAD using the PerCP-Cy5.5 channel and a region for live cells was defined. Non-infected cells were used as a control. Q1—early apoptotic cells; Q2—viable cells; Q3—late apoptotic cells; and Q4—necrotic cells.

Apoptosis begins among all tested human glioblastoma cell lines at 24 h p.i. and probably develops in a cascade manner (Figure 2). This suggests the occurrence of early and late apoptosis. It is assumed that viral replication initiates cell death and lysis, accompanied by the release of progeny viruses that infect subsequent cells. Infected murine GL261 cells were destroyed before the 24 h time-point p.i. (Video S1). 

In the ADSCs cell line (used as the virus carrier), the apoptosis test showed cells to remain viable up to 24 h p.i., without features of membrane damage. Within 48 h p.i., the ADSCs acquired characteristics of necrotic cells, which indicates extensive damage to the cell membrane and mechanical disintegration. In contrast, non-permissive bEnd.3 cells used for making an artificial BBB do not undergo apoptosis at all, but the presence of necrotic cells was observed after 48 and 72 h p.i.

**Figure 3:**
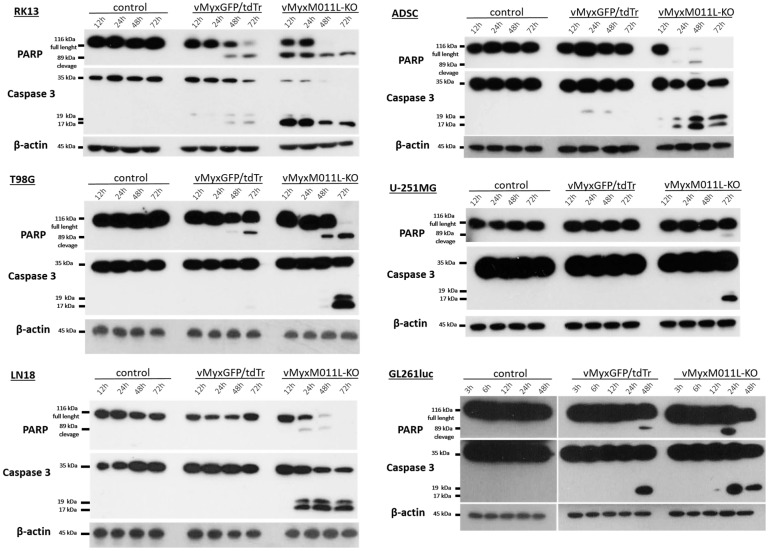
Expression of apoptotic proteins by Western blot analysis of lysates obtained from cell cultures infected (MOI = 5) with vMyx-EGFP/tdTr or vMyx-M011L-KO/EGFP constructs. Lysates were prepared from whole cell extracts after 12, 24, 48 and 72 h p.i. (RK-13, ADSCs, T98G, U-251 MG and LN18) and after 3, 6, 12, 24, and 48 h p.i. (GL261luc; control samples run on separate gel, same experiment). Full-length (116 kDa) and cleaved forms (89 kDa) of poly ADP-ribose polymerase (PARP), as well as endogenous levels of the full length caspase-3 (35 kDa) and the large fragment of caspase-3 resulting from cleavage (19/17 kDa) were assessed by immunoblotting using β-actin as a loading control. See also Figure S1.

Western blot analysis (Figure 3) of protein expression showed the presence of protein forms associated with induction (active caspase 3) and ongoing apoptosis (cleaved form of poly (ADP-ribose) polymerase (PARP) in all cell lines infected with the vMyx-M011L-KO/EGFP construct as well as with the vMyx-GFP/tdTr reporter construct. The presence of these protein forms indicates the occurrence of apoptosis associated with the mitochondrial pathway and was identified in the early stage post infection (after 12 h for RK13, 24 h for GL261-luc and ADSCs, and after 48–72 h for human glioblastomas). 

The apoptotic effect after infection with the vMyx-M011L-KO construct is much stronger and faster than that of the vMyx-GFP/tdTr reporter construct; the differences in apoptosis induction were noted from 36 h (RK13 control line) and from 24 h (human glioblastoma lines).

### 2.3. Myxoma Virus Construct-Infected ADSCs Migrate through Artificial Blood–Brain Barrier (BBB) and Allow Infection of Target Glioma Cells

**Figure 4:**
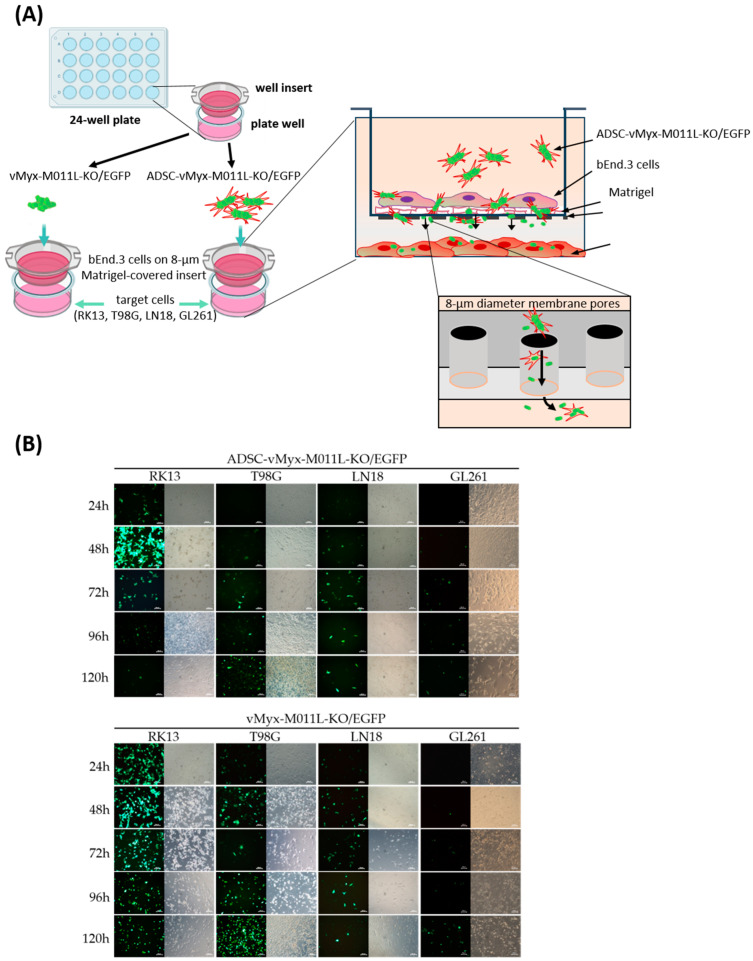
In vitro blood–brain barrier (BBB) migration studies. (**A**) Schematic representation of the barrier formed by plating bEnd.3 cells (murine endothelial cells isolated from endothelioma) on inserts of the 24-well plate (Matrigel^®^-covered 8 µm pore inserts). Studies of virus migration through the BBB were started when transendothelial electrical resistance (TEER) of the BBB (measured with an ohmmeter) reached ca. 20 Ω × cm^2^. The virus migration assay was performed by administering three equivalent doses of ADSCs infected with vMyx-M011L-KO/EGFP (MOI = 5) or unshielded virus every 24 h on pre-prepared inserts. (**B**) The migration was assessed within a 24–120 h period after the addition of the virus construct or virus-loaded ADSCs (microscopic observation in visible light and using the green fluorescence channel (EGFP), ZEISS AxioVert microscope, 20× magnification, scale bar = 100 µm).

The proposed further systemic therapy assumes the ability of the therapeutic platform to penetrate the physiological blood–brain barrier (BBB). We verified its ability to overcome the model blood–brain barrier formed by cultured bEnd.3 cells. Changes in transendothelial electrical resistance (TEER) of the system were examined. The artificial BBB was formed on Matrigel^®^-coated polystyrene inserts (8 μm diameter pores) placed in the wells of a culture plate (24-well, Corning, Corning, NY, USA). The resistance values (ca. 20 Ω × cm^2^) measured on both sides of the bEnd.3 cell layer were assumed to indicate a sufficient degree of artificial barrier cohesion. Suspensions of ADSCs infected with the vMyx-M011L-KO/EGFP construct or the virus alone were then applied on the apical surface of the inserts (Figure 4A). The inserts were replaced after 24 h or 48 h, depending on the experiment. Cultures of RK13 (positive control), human glioma (T98G, LN18) or mouse glioma (GL261) cells cultured at the bottom of the well served as the final “therapeutic” target. The migration capacity through this artificial barrier (“bioavailability” of the tested therapeutic systems) was assessed after 24–120 h post addition of virus-infected ADSCs or the virus itself. Fluorescence- and light microscopy-based evaluation of the established target cells was performed (Figure 4B). 

Three doses of ADSC-shielded viral construct were applied onto the BBB, since with a single dose of the virus overgrowth of the target cell cultures occurred. The formation of single foci of infection in the target cells’ layer was noticed for both therapeutic schemes applied (virus alone and ADSC-shielded virus) in the early stages (24 h post the first dose) of the experiment (Figure 4B). Initially, the cytotoxic effect of the unshielded virus was stronger and faster. After three doses (with a change of insert at 24 and 48 h), clear infection of the target glioma cells was observed for both therapeutic variants (Figure 4B). The infection developed most rapidly and intensely in the RK13 cell line (positive control), as expected. In the case of human glioma culture, the first signs of infection were observed after 24 h, which over time led to the formation of viral outbreaks that ultimately reduced cell viability. In murine glioma culture (the MYXV-sensitive cell line in which the construct does not replicate), the first EGFP foci were observed after 48 h and became slightly more were apparent with time elapsed. Infected cells ultimately were destroyed in all cultures of the cell lines tested. The most intense decrease in viability was observed for GL261 at 120 h p.i. using three doses of MYXV and virus-loaded ADSCs (Figure 4B). No clear difference was observed between unshielded and ADSCs-shielded virus for either cell line tested. At the end point of infection of target cells, i.e., at 120 h, the cytotoxic effect was comparable for the RK13 and T98G cell lines (100%) and for the LN18 and GL261 lines (90–95%). The experiment confirmed the possibility of the migration of functionally active vMyx-M011L-KO/EGFP virus through an endothelial cell barrier, when administered directly to bEnd.3 cells and when released from the ADSC cell carrier.

### 2.4. Myxoma Virus Construct Inhibits Lesion Formation and Prolongs Survival in an Immunocompetent Orthotopic Syngeneic Murine Glioma Model 

**Figure 5:**
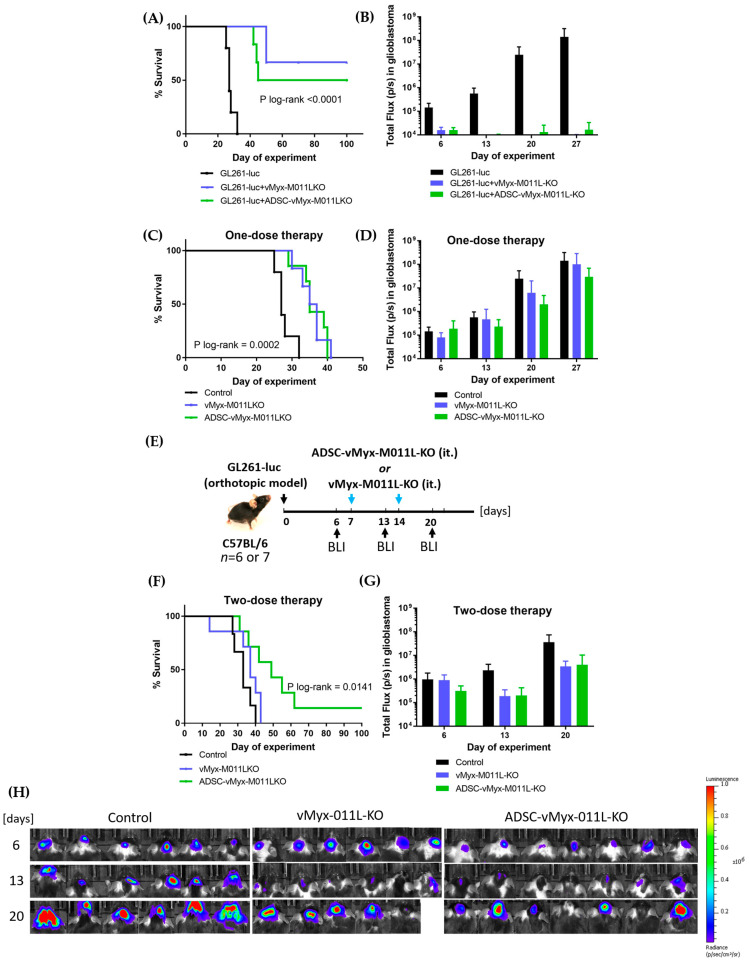
The therapeutic effect on experimentally-induced murine glioma lesions following intracranial treatment with shielded or unshielded vMyx-M011L-KO/EGFP constructs. (**A**) Inhibited lesion formation and prolonged survival: mice with orthotopically administered GL261-luc glioma cells were subjected to simultaneous (consecutive) injection of either unshielded or ADSC-shielded vMyx-M011L-KO/EGFP knockout construct. (**B**) ROI-based bioluminescence (BLI) analysis of total photon flux in mouse heads. IVIS-detected signal at 4 time points following GL261-luc implantation (mean +/− SD from 6 mice/group). (**C**) One-dose therapy: mice with similar intracranial BLI randomly divided (6th day post inoculation) into groups were injected intratumorally (day 7) with unshielded vMyx-M011L-KO/EGFP construct or ADSC-shielded construct. (**D**) One-dose therapy: ROI-based BLI analysis of total photon flux in mouse heads: IVIS-detected signal at four time points following GL261-luc implantation (mean +/− SD from 6 mice/group). (**E**) Timetable of two-dose therapy. (**F**) Two-dose therapy: mice with similar intracranial BLI randomly divided (6th day post inoculation) into groups were injected intratumorally (days 7 and 14) with unshielded vMyx-M011L-KO/EGFP construct or ADSC-shielded construct. (**G**) Two-dose therapy: ROI-based BLI analysis of total photon flux in mouse heads: IVIS-detected signal at three time points following GL261-luc implantation (mean +/− SD from 6 or 7 mice/group). (**H**) IVIS-detected signal following two-dose therapy: ROI-based analysis of total photon flux in mouse heads. BLI expressed as radiance (photons/sec/cm^2^/sr). Single radiance scale shown to cover the whole span of bioluminescence. The data show mean ± SD of two independent experiments.

To obtain the proof of principle of the therapeutic platform usefulness, we first examined tumor growth dynamics following co-injection (1:1 ratio) of either GL261-luc cells and ADSCs pre-infected with the vMyx-M011L-KO/EGFP construct, or co-injection of GL261-luc cells and vMyx-M011L-KO/EGFP construct alone. Injections were administered into the brain right hemisphere of immunocompetent mice using a stereotactic system. Dynamics of tumor growth were followed by measuring the bioluminescence signal (BLI) and survival of the treated mice. The median survival (Figure 5A) was found to be significantly longer in mice injected with GL261-luc cells and the knockout construct (72.5 days), as compared to the control (GL261-luc injection only, 27 days). However, the median survival of animals co-implanted with GL261-luc cells and, concomitantly, with vMyx-M011L-KO/EGFP, was not reached, as the experiment was arbitrarily terminated after 100 days (log-rank, *p* = 0.0001; Figure 5A). The BLI signal (Figure 5B) detected in mice treated with GL261-luc alone was clearly increased at the time points examined, indicating continued tumor growth in the control group. In contrast, groups of mice treated with GL261-luc cells and co-injected with the unshielded vMyx-M011L-KO/EGFP construct, or the ADSC-shielded knockout virus construct, showed very low BLI signal at the same time points. In addition, we observed disease-free progression in 67% of the animals given the unshielded virus and 50% of the animals given the ADSC-shielded virus, suggesting a marked inhibition of tumor growth in these groups.

We then tested the effect of delivering the examined therapeutic platform in an established murine model of orthotopic glioma brain lesions. Following orthotopic implantation of GL261-luc cells into the right brain hemisphere of mice, 7 days later we performed a single stereotactic injection into the tumor lesion using the vMyx-M011L-KO/EGFP construct alone or using the construct-infected ADSC (Figure 5C,D) To assess the tumor size on days 6, 13, 20 and 27, we tested the BLI signal. On day 6, mice with similar BLI signals were divided into study groups. In this single-dose therapy, slightly slower tumor growth was observed as compared to control (Figure 5D); the median survival (35 days for vMyx-M011L-KO/EGFP and 36 days for vMyx-M011L-KO/EGFP-infected ADSC) was found to be only slightly different from the median survival of the control group (27 days; Figure 5C). 

Based on the above result, we assumed that a two-dose therapeutic experiment (Figure 5E–H) might increase the therapeutic efficacy and yield longer animal survival. No therapeutic advantage over control was observed for unshielded viral construct double administration. The median survival was 37 days and, based on BLI signals, growth of the tumors was slower (Figure 5G,H). On the other hand, two-dose therapy (Figure 5F) using the ADSC-shielded viral construct was more beneficial in terms of prolonging survival (median of 45.5 day, *p* log-rank = 0.0186). Additionally, we observed disease-free progression in 12.5% of the animals (Figure H), suggesting that repeated dosage of ADSCs infected with vMyx-M011L-KO/EGFP can slow down or stop tumor growth. The results of this therapeutic experiment thus show that glioma lesions in this orthotopic murine model are indeed susceptible to local treatment with the ADSC platform ferrying the proapoptotic vMyx-M011L-KO/EGFP construct. Viral particles released in situ from infected ADSCs can successfully infect and kill glioma cells. As repeated supply of the examined myxoma construct to glioma lesions is more effective, this speaks in favor of further exploration of the system using systemic intraarterial delivery. 

## 3. Discussion

Unique therapeutic challenge such as slow dividing neurons and glial cells, as well as recurrence driven by a highly treatment-resistant stem cell-like compartment, allow glioblastoma (GBM) tumors to evade chemotherapy and radiation therapy as the latter focuses on killing rapidly dividing cells [6]. The outcome of traditional treatment has remained static and poor for decades. Current therapeutic options, such as temozolomide, carmustine, and lomustine; hypofractionated-dose radiotherapy; bevacizumab; and a handful of investigational targeted drugs continue to result in dim survival prognoses. 

Therefore, novel therapeutic strategies are urgently needed to surpass the current obstacles in GBM therapy. Oncolytic viruses (OV), weakly pathogenic viruses that can selectively infect, replicate in cancer cells and eventually lyse them without damaging normal cells have been emerging as a powerful treatment tool against GBM. Several viruses are being investigated (ca. 30 current clinical trials) including herpes simplex, adenovirus, measles virus, parvovirus, reovirus poliovirus and zika virus [19]. Delytact, a third generation (triple-mutated) recombinant oncolytic herpes simplex virus type 1, is the first oncolytic tool conditionally approved in Japan to treat certain recurrent gliomas. To date, several other clinical trials have been initiated for oncolytic viruses to improve the treatment of GBM [20]. Examples include adenovirus, herpes simplex virus, reovirus, parvovirus, measles virus, poliovirus, vaccinia virus and Newcastle disease virus. Ongoing or completed trials have used modified HSV constructs such as G207, MVR-C252, M032 and C134, as well as adenoviral constructs combined with immune checkpoint blockade [21].

Myxoma virus biology also offers a therapeutic vista for glioma treatment. This oncolytic virus encodes proteins that can modulate host survival pathways to override programmed cell death. The genetic construct examined in this study, vMyx-M011L-KO/EGFP, has shown efficacy as a potential GBM treatment [5]. Deletion of the M011L gene is sufficient to trigger apoptosis of both human and murine brain tumor initiating cells (BTICs) thought to shoulder the major responsibility for tumor recurrence. We examined how this pro-apoptotic construct affected cultures of human and murine glioma cell lines in vitro. We show here that all tested glioma cell cultures were indeed destroyed by the engineered myxoma construct (Figure 1, Figure 2 and Figure 3). Of note, the dynamics of GL261 murine glioma cell death differed from the other tested glioma cell lines. The murine line, shown to be semi-permissive to both wild-type myxoma virus and the therapeutic construct, was particularly sensitive to cytotoxic effects, as evidenced by the MTS results and microscopic observation. The cause of cell death seen early post infection (3–12 h p.i.) is not clear (Video S1).

However, all of these viruses have been so far administered into the brain through stereotactic brain surgery. Intratumoral administration of drugs to the brain is extremely complex and expensive. The difficulties are compounded by the risk of intracranial bleeding and neurological deficits. On the other hand, the benefits associated with intravascular (systemic) injection of anti-glioma drugs are rather undisputable. Albeit technically difficult in rodents, endovascular techniques would be much more straightforward in the clinic [22].

A formidable obstacle to systemic drug delivery to the brain is, however, the presence of the blood–brain barrier (BBB), regulating the transit of ions, small molecules and macromolecules to the brain and protecting it from inflammation, toxins and injury [23,24]. The inflammatory and pro-angiogenic character of the glioma microenvironment does affect the permeability of the BBB [19]. Disease progression of low-grade to high-grade glioma is characterized by increased vascularization associated with BBB disturbances. BBB alterations are most prominent in glioblastoma multiforme [25]. 

Successful systemic delivery of oncolytic virotherapeutics might indeed unleash their clinical potential [16]. It does face another natural limitation due to a knockdown in infectivity [16]. Also, since the physical passage of oncolytic virus across the BBB is rather stringent [19], we wondered if the myxoma virus construct lacking the M011L gene, shielded by a Trojan horse-like ADSC carrier, could be successfully delivered through an artificial BBB barrier as a prerequisite for systemic delivery. In conjunction with the ability of virus-loaded ADSCs to seek out all brain tumor cells and deliver oncolytic myxoma virus, a systemic approach would be of great therapeutic value [26]. Such a proof-of-concept would also indicate that OV administration via stereotactic brain surgery might be obviated with this platform and would suggest its relevance for further development as a systemic therapeutic. 

The use of MSCs to ferry drugs to the brain across the BBB appears to be a dynamic strategy. Due to the size and ability of MSCs to adopt a spindle-like shape, they may be small enough not to occlude microvascular structures of the BBB, move through narrow areas and be bound to the surface of capillary endothelial cells via the interaction of adhesion molecules with endothelial surface receptors, as well as due to the so-called fluid shear stress pushing MSCs toward the endothelial cell surface [17,27]. 

Allogeneic MSCs are hypoimmunogenic cells easily obtained from patients without ethical concerns. They can be administered to allogeneic hosts, making them versatile and universal cargo vectors. The ADSC vehicle protects the viral load during transit to the target tumor tissue from being captured by the host immune system cells, bloodstream components and resident macrophages, and ultimately, neutralization. In general, MSCs can migrate to cancer foci due to a chemoattractant gradient created by cytokines secreted in the tumor milieu. ADSCs show negligible tropism for normal brain cells; although a viral cargo (including progeny viral particles) can subvert their cellular metabolism, the viral cargo has no significant impact on the functional characteristics of the carrier cells [7,8,28]. The first report of the use of MSCs for the treatment of GBM was in 2004 [29]. MSCs show tropism to and localize in glioma tumors, likely because their stromal milieu recalls non-healing wounds (“tumor, a wound that does not heal”). This feature of MSCs, together with active escape of the viral cargo from host immune system surveillance, boosts the destructive power of oncolytic viruses. With precautions and barriers to overcome, improvements in engineering of viral constructs and MSCs move toward developing novel Trojan horse-like solutions of MSC-mediated delivery of oncolytics into a viable therapeutic option. Nevertheless, human MSCs as carriers for drug delivery have not yet received clinical approval. 

MSCs should be administered intraarterially, possibly via the internal carotid artery to eliminate the so-called first-pass effect in the lungs, resulting from the size of the MSCs and interactions of their adhesion molecules with endothelial receptors.

The question was whether i.v. delivery of virus-loaded ADSCs to glioma lesions would be hindered by the BBB presence. Selective homing of fluorescently labeled MSCs to glioma intracranial xenografts was demonstrated in mice as early as 2005, following intracarotid injection of fluorescent human MSCs. These MSCs were then found exclusively within the brain tumors regardless of whether the cells were injected into the ipsilateral or contralateral carotid artery [30]. Human mesenchymal stem cells (hMSCs) were also used for delivering to human glioma xenografts a genetically engineered conditionally replicative adenovirus (Delta24-RGD). Virus-infected hMSCs selectively localized to glioma xenografts and released Delta24-RGD, which subsequently infected glioma cells [22]. MSCs carrying this construct were also demonstrated to home to glioma stem cells (GSC), tumor-initiating cells responsible for treatment failures, with the TGF-β/TGFβR axis shown as a mediator of the tropism of BM-hMSCs for GSCs [31]. 

Our own data show that the delivery of ADSCs infected ex vivo with a myxoma knock-out construct to the luminal side of the model BBB results in translocation of the therapeutic cargo to the abluminal side of the barrier. Subsequent transduction of targeted glioma cells grown on the bottom of culture plate wells (Figure 4), distally from the artificial BBB, was the outcome of either virus release from ADSCs on the distal side of the barrier, and/or contact of virus-loaded ADSCs with targeted glioma cells, and/or virus release with translocation from the luminal side of the barrier. The release modes could be complementary and all resulting in transduction. Passage of stem cells in vivo through the actual BBB into the parenchyma of a brain lesion-bearing animal is likely to occur via fenestrae forming in the endothelial cells’ layer tight junctions [17].

ADSCs infected with vMyx-M011L-KO/EGFP are permissive for myxoma virus replication and support single and multiple rounds of replication, leading to productive infection. This is a crucial aspect of this platform’s usefulness since myxoma virus replication must assure successful cross-infection of targeted tumor cells. The timing of intravascular delivery of MSCs in relation to their infection ex vivo is likely critical for optimizing viral yield in vivo. By delivering MSCs 24–48 h after infection with oncolytic virus, robust tumor infection can be achieved. Completion of the myxoma virus replication cycle is the doomsday for the MSCs. This protects against any pro-tumorigenic risks of the MSCs and stimulates an antitumor immune response [22].

We further wondered if the tested viral platform could possibly improve the elimination/reduction of experimental murine glioma lesions in vivo. Syngeneic C57BL/6 mice with induced orthotopic GL 261 lesions recapitulate pertinent histopathologic attributes of the human glioma; the model also maintains an intact immune microenvironment needed for optimal efficacy of the oncolytic strategy. Expression of luciferase in the GL261 glioma model enables monitoring of intracranial tumor growth [32]. Our in vivo study of ADSCs pre-loaded ex vivo with vMyx-M011L-KO and injected stereotactically into the tumor bed of GL261-bearing mice showed that twice-repeated intralesional administration conferred a survival advantage over a double dose of the unprotected virus and an advantage over one-dose administration of both unprotected and protected virus (Figure 5). ADSCs can thus act as “Trojan horses” capable of delivering myxoma virus to glioma lesions. 

Taken together, the ability of a pro-apoptotic myxoma construct ferried by an ADSC carrier to infiltrate the artificial blood–brain barrier (BBB), as evidenced by infection of glioma cells cultured below the barrier (distally), points to the timely release of viral particles from the carrier. The therapeutic benefit of this platform (slowed-down progression of GL261-luc and prolonged survival of mice) noted in the twice-repeated intralesional treatment of mice highlights the advantage of temporally shielding the virus in transit to its destination target. The reduced tumor burden and increased survival are likely due to cell death induced directly by vMyx-M011L-KO/EGFP, as well as by immune mediators. 

In view of possible improvements in ADSC homing to glioma lesions (e.g., by exploring overexpression of the chemokine receptor CXCR4 and the presence of its ligand CXCL12 in glioma lesions), as well as likely additional arming of the myxoma construct with novel therapeutic transgenes, the studied platform could be developed into a powerful tool for a non-invasive approach to destroying glioma lesions. This approach involves microsurgical injection of therapeutic cargo into the internal carotid artery while occluding the lumen of the external carotid artery with a special catheter. This set-up allows therapeutic cargo injection as close as possible to the luminal side of the BBB.

Furthermore, its use in combination with various other treatment modalities (e.g., radiotherapy [33]) can reasonably be expected to produce synergistic therapeutic effects.

## 4. Materials and Methods

### 4.1. Cell Culture 

The following cell lines were used: RK13 (rabbit kidney epithelium, ATCC); ADSC (adipose derived mesenchymal stem cells, isolated, phenotyped, and cultured as described in [8]); GL261luc (murine glioblastoma expressing luciferase gene, a gift from Dr. Ryszard Smolarczyk); LN18 and U-251MG (human glioblastoma, a gift from Dr. Agnieszka Szurko); T98G (human glioblastoma, ATCC) and bEnd.3 (murine SV129 brain endothelioma, ATCC). ADSCs were maintained in MEM (Sigma-Aldrich, St. Louis, MO, USA) with 10% human platelet lysate (EMD Millipore, Burlington, MA, USA), heparin (Polfa, Warszawa, Poland) and 1% antibiotics (Penicillin-Streptomycin, Sigma-Aldrich, St. Louis, MO, USA). The remaining cell lines were cultured in DMEM (Sigma-Aldrich, St. Louis, MO, USA) with 10% fetal bovine serum (origin – Brazil; EURx, Gdansk, Poland) and 1% antibiotics. Cultures were maintained at 37 °C, 5%CO_2_; confluent cultures were passaged at a split ratio of 1:5~1:10. All cell lines were tested for the presence of pathogens.

### 4.2. Viruses 

The vMyx-EGFP/tdTr myxoma construct was derived from the wild-type Lausanne strain of myxoma poxvirus. The vMyx-M011L-KO/EGFP construct with *M011L* gene knockout was described previously [5]. Both viruses were propagated and titrated as described [7].

### 4.3. Viral Replication 

The ability of both viral constructs to replicate in the studied cell lines was examined by generating single-step and multiple-step virus growth curves. Generation of a single-step growth curve (at MOI = 5) was described previously [7]. A multi-step growth curve was performed (at MOI = 0.02) by extending the propagation time to 72 h (cell material collected at time points: 0, 24, 48 and 72 h).

### 4.4. Infectivity 

Infection ability of viral constructs was assessed visually by microscopy. Cell lines were treated with vMyx-M011L-KO/EGFP at MOI = 5. Next, cell cultures were stained 24 h post-infection with DAPI (D9542, Sigma, Tokyo, Japan,) and examined using a confocal microscope (Zeiss LSM 710 workstation, Carl Zeiss AG, Jena, Germany) at 20× magnification.

### 4.5. Permissiveness of Cell Lines 

Cells were seeded in 6-well plates (4 × 10^5^ cells/well) and infected 24 h later with vMyx-M011L-KO/EGFP (MOI = 5). Only adherent cells were further analyzed. Cells were collected (at 12, 24 and 48 h time points in the case of RK13, ADSC, bEnd.3 and Gl261-luc cell lines, or at 12, 24, 48 and 72 h time points in the case of human glioblastoma lines. Uninfected cells served as controls. Prior to flow cytometric readouts, 7-aminoactinomycin D (7-AAD) was added to the cells and EGFP protein fluorescence was measured (BD FACSLyric™), Becton Dickinson, New York, NY, USA).

### 4.6. Cell Viability 

Viability of cell lines infected with vMyx-M011L-KO/EGFP was assessed by MTS assay. Cells were seeded (1 × 10^4^/well) in a 96-well plate and infected 24 h later using four increasing MOI values (0.1, 1, 5 and 10). Mitochondrial activity was quantified 24 and 48 h post infection (RK13, ADSC, bEnd.3 and Gl261luc) or 24, 48 and 72 h post infection (LN18, T98G and U-251MG) after addition (1.5 h incubation) of MTS reagent (Promega, Madison, WI, USA) at λ = 490 nm.

### 4.7. Apoptosis Assay 

Cells were treated as in the permissiveness assessment. Collected cells were stained with Annexin V-Pacific Blue™ (BioLegend, San Diego, CA, USA) and 7-AAD for 15 min, then analyzed by flow cytometry (BD FACSLyric™) to determine the expression of Annexin V.

### 4.8. Western Blot Analysis 

Analyses were performed using cell lysates from RK13 (control), ADSC, GL261-luc (mouse) as well as U-251MG and T98G (human) glioblastoma lines. Cultured cells were infected (MOI = 5) with the vMyx-GFP/tdTr reporter construct or with vMyxM011L-KO/EGFP and harvested after 12, 24, 48 and 72 h (RK13, ADSC, U-251MG and T98G cell lines) or after 3, 6, 12, 24 and 48 h (GL261-luc). Cell lysates were prepared from total cellular material (dead/detached cells and live cells) and trypsinized cells. Primary rabbit antibodies for PARP (1:500), Caspase 3 (1:1000) and β-actin (1:10 000) and a secondary anti-rabbit antibody (1:3000) were used (Cell Signaling, Beverly, MA, USA). Proteins were detected on the membrane by chemiluminescence (Thermo Scientific, Waltham, MA, USA) and visualized on X-ray film.

### 4.9. Artificial Blood Brain Barrier Studies 

To generate the BBB study model, bEnd3 cells were seeded (2.5 × 10^4^) on the apical surface of the Transwell insert membrane (Falcon^®^ Permeable Support for 24-well Plate with 8.0 µm Transparent PET Membrane, Corning B.V., USA; 353097) coated with Matrigel™ (Discovery Labware, Bedford, MA, USA) 2% solution. Twenty-four hours after seeding the bEnd.3 cells, transendothelial resistance (TEER) values [Ω × cm^2^] of the system (see Figure 4A) were measured using an Epithelial Voltmeter (EVOM) with Endohm-12 cell electrodes (World Precision Instrument, Sarasota, FL, USA). The TEER value of the empty inserts was later subtracted from the measured TEER in each study model adapted as in [34]. After the TEER value for the cell barrier formed on the insert reached ca. 20 Ω × cm^2^ (appr. 24 h), human glioma cells (T98G and LN18), mouse glioma cells (GL261), or normal rabbit epithelial cells (RK13, control) were seeded on the well bottom (5 × 10^4^/well) and the whole plate was incubated for 8 h. Then, ADSCs infected (24 h earlier; MOI = 5) with the vMyxM011L-KO-EGFP construct or the viral construct alone were applied onto the bEnd.3 cell layer. The first dose was added on day 0, the second after 24 h, and the third after 48 h. The migration was monitored microscopically (20× magnification) in visible light and by applying a green fluorescence (EGFP) channel (ZEISS AxioVert 5 microscope, Carl Zeiss AG, Jena, Germany) throughout the 24–120 h period following the input of virus-loaded ADSCs or virus alone.

### 4.10. Animal Care 

C57Bl/6NCrl immunocompetent mice (females, 6–8 weeks old, Charles River Breeding Laboratories, Wilmington, MA, USA) were used for the in vivo experiments. Animals were housed in the Animal Facility (on site) under sterile conditions using individually ventilated cages (Allentown Caging Equipment, Allentown, NJ, USA) on a controlled 12 h/12 h light:dark cycle, with free access to water and a standard pathogen-free Altromin 1314 diet. All breeding procedures and experiments were performed in accordance with European Union law and institutional standards. Every effort has been made to minimize animal suffering. The animal study protocol was approved by the Local Ethics Committee for Animal Testing at the Medical University of Silesia in Katowice (Approval No. 15/2022). 

#### Animal Therapy 

All injections were orthotopic and performed using stereotactic equipment. In the initial experiment, a group of mice were injected with GL261-luc glioblastoma cells (1.5 × 10^5^/2 µL PBS^−^ mouse). Some mice were consecutively injected (first group) with vMyx-M011L-KO/EGFP (7.5 × 10^5^ FFU/2 µL PBS^−^/mouse; *n* = 6); or (second group) with ADSCs (1.5 × 10^5^ cells/2 µL PBS^−^/mouse; n = 6) infected (MOI = 5) on the preceding day with vMyx-M011L-KO/EGFP. Control mice received glioma cells as above, followed by injection of PBS^−^ only (2 µL/mouse; *n* = 5). 

In the subsequent therapeutic experiments, tumors were first established in the recipient mice by injecting on day zero GL261-luc cells (1.5 × 10^5^/4 µL PBS^−^/mouse) into the right brain hemisphere. Six days following inoculation, mice with similar bioluminescence IVIS signals were randomly divided into groups and therapy was initiated on day 7. In single-dose treatment, the tumors were injected with either vMyx-M011L-KO/EGFP (7.5 × 10^5^ FFU/2 µL PBS^−^/mouse), or with ADSCs (1.5 × 10^5^ cells/4 µL PBS^−^/mouse; *n* = 7) that had been infected (MOI = 5) on the preceding day using the therapeutic construct, or with PBS^−^ only (2 µL/mouse; *n* = 5). For two-dose therapy (days 7 and 14), the mice bearing tumors were treated as above for single therapy. Then, the therapeutic interventions were additionally repeated on day 14: the tumors were injected with either vMyx-M011L-KO/EGFP (7.5 × 10^5^; n = 7 and 1.5 × 10^5^ FFU/2 µL PBS^−^/mouse; *n* = 7), ADSCs (1.5 × 10^5^ cells/4 µL PBS^−^/mouse; *n* = 7), or PBS^−^ (2 µL/mouse; *n* = 6).

### 4.11. Bioluminescence Imaging 

The dynamics of tumor growth were followed using an IVIS Lumina II equipped with Living Image 3.2 software (PerkinElmer, Waltham, MA, USA). The animals were injected ip with d-luciferin (15 mg/mL; VivoGlo Luciferin; Promega, Madison, WI, USA) suspended in 200 μL PBS^−^ and sedated using isoflurane (2%). Bioluminescence signals in mice burdened with GL261-luc were acquired on day 6, 13, 20 and 27 (for the initial experiment and one-dose therapy) and on day 6, 13 and 20 (for two-dose therapy) following implantation of GL261-luc cells (mean +/− SD from 5–7 animals/group). With the BLI data acquired, regions of interest (ROIs) were determined and expressed as total flux (p/s).

### 4.12. Statistics 

Graphs were plotted and analyses of statistical differences performed using GraphPad Prism software (Version 7). The results were analyzed using one- or two-way ANOVA followed by Tukey’s multiple comparison test. To ensure that the data meet the assumptions of parametric significance tests, Bartlett’s test was run. Kaplan–Meier survival curves were examined using the Mantel–Cox test. Data are presented as bars showing mean ± standard deviation. *p*-values below 0.05 were judged statistically significant.

## 5. Conclusions

An MYXV knock-out construct lacking the gene for anti-apoptotic protein M011L pro-actively induced apoptosis of cultured glioma cells that are normally resistant to apoptosis induction. A viral construct-infected ADSC platform was shown to be capable of delivering and releasing virus that translocated through an artificial blood–brain barrier (BBB) and retained the capability to infect cultured glioma cells. The viability of ADSCs infected with the proapoptotic myxoma construct appeared to be more than sufficient (”therapeutic window”) for successful transfer of the oncolytic agent to glioma lesions. Local injection of ADSCs ferrying the myxoma oncolytic cargo slowed down growth of orthotopic murine GL261-luc experimental lesions and prolonged animal survival. This suggests further development is warranted of this oncoviral platform into a prospective tool for non-invasive (systemic) targeting and elimination of glioma lesions. 

Our immediate research quest involves exploring microsurgical delivery of our therapeutic platform to glioma-bearing mice via the internal carotid artery. This is a much less invasive approach to treat CNS lesions than direct interference with the glioma. Since combining innovative strategies might positively impact progress in glioma research, we also plan to explore the systemic oncolytic virotherapy platform together with a BBB-crossing innovative chemotherapeutic. 

## Data Availability

Data is contained within the article and Supplementary Materials.

## Supplementary Materials

The following supporting information can be downloaded at: https://www.mdpi.com/article/10.3390/ijms252011225/s1.
