# Supplementary figures and images for: Adipose-Derived Stem Cells as Carrier of Pro-Apoptotic Oncolytic Myxoma Virus: To Cross the Blood–Brain Barrier and Treat Murine Glioma
Source: Int J Mol Sci. 2024 Oct 18;25(20):11225. doi: 10.3390/ijms252011225 (PMC11508294; doi:10.3390/ijms252011225)

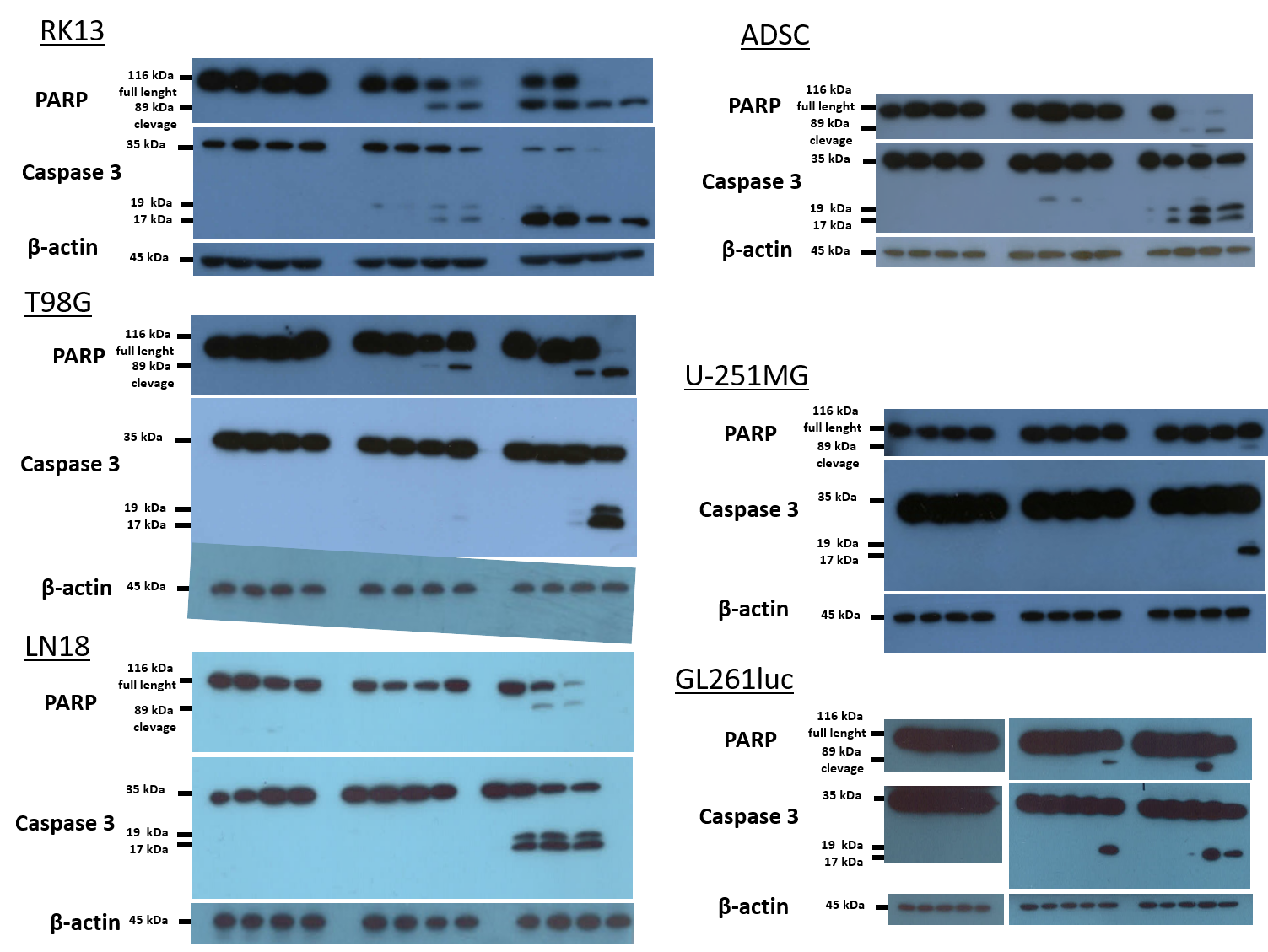

Supplement: Supplementary file 1 [file ijms-25-11225-s001.zip › Figure 1S.tif]
